# COVID-19-induced myocarditis in patient with anomalous origin of the left circumflex artery from the right coronary artery: a case report

**DOI:** 10.1590/1677-5449.202102132

**Published:** 2022-11-25

**Authors:** Majed Khalil, Batoul Danash, Dany Akiki, Nabil Khoury

**Affiliations:** 1 Hôpital Libanais Geitaoui – HLG, Beirut, Lebanon.; 2 Lebanese University, Faculty of Medical Sciences, Beirut, Lebanon.

**Keywords:** COVID-19, right coronary artery, anomalous circumflex artery, coronary angiography, viral-induced myocarditis, case report, COVID-19, coronária direita, artéria circunflexa anômala, angiografia coronária, miocardite induzida por vírus, relato de caso

## Abstract

The prevalence of coronary artery anomalies has been increasing due to the increasing usage of coronary angiography. There is a paucity of literature concerning management of viral-induced myocarditis in patients with anomalous coronary artery. We present a very unusual case of a 44-year-old man with anomalous origin of the left circumflex artery from the proximal ostium of the right coronary artery who was admitted for COVID-19-induced myocarditis. He presented with signs of heart failure and coronary angiography revealed the left circumflex artery with a separate ostium originating from the proximal right coronary artery. He was treated medically with Bisoprolol, Perindopril Arginine, Rivaroxaban, and Furosemide. His condition improved rapidly and he resumed regular life within 1 month. Coexistence of cardiac disease such as viral-induced myocarditis with an underlying anomalous origin of the coronary artery is challenging to spot and can lead to worse outcomes in case of misdiagnosis and inaccurate management.

## Resumo

A prevalência de anomalias da artéria coronária vem aumentando devido ao uso crescente da angiografia coronariana. Há uma escassez de literatura sobre o manejo da miocardite induzida por vírus no contexto de artéria coronária anômala. Apresentamos um caso incomum de um homem de 44 anos com origem anômala da artéria circunflexa esquerda do óstio proximal da artéria coronária direita admitido por miocardite induzida por COVID-19. O paciente apresentava sinais de insuficiência cardíaca, e a cineangiocoronariografia revelou artéria circunflexa esquerda de óstio separado originando-se da artéria coronária direita proximal. Ele foi tratado clinicamente com bisoprolol, perindopril arginina, rivaroxabana e furosemida. Sua condição melhorou rapidamente, e o paciente recuperou uma vida normal em 1 mês. A coexistência de doença cardíaca, como miocardite induzida por vírus com uma origem anômala subjacente da artéria coronária, é difícil de detectar e pode levar a resultados piores em caso de diagnóstico incorreto e manejo impreciso.

## INTRODUCTION

The most common coronary artery anomaly is ectopic origin of the left circumflex artery.^1^ It can originate from the proximal part of the right coronary artery or from a separate ostium within the right sinus. Prevalence is estimated at 0.24 to 0.46% of all patients.^2^^,^ 3 Overall, 0.2 to 1.3% of coronary angiographies show evidence of anomalous coronary arteries.^4^ The association of these anomalies with coronary artery disease and of these findings with substantial heart failure mandates their identification. In this report, we present a rare case of a 44-year-old man with anomalous left circumflex artery originating from the proximal right coronary artery who presented with viral-induced myocarditis with heart failure. Coexistence of COVID-19-induced myocarditis with abnormal anatomy of the circumflex artery is a rare condition in which implementation of the best treatment modality and prediction of disease progression and outcomes are challenging.

The protocol was approved by the HLG institutional Ethics Committee.

## CASE DESCRIPTION

A 44-year-old man presented at the emergency room (ER) with dyspnea, lower limb edema, and limited ability to perform physical exercise, which had been worsening over the past few months. The patient had a history of hypertension, uncontrolled diabetes mellitus type 2 with a most recent HbA1c value of 9.4%, and COVID-19 pneumonia one month before presentation. He had no significant past surgical or family history. The list of home medications was Vildagliptin/Metformin (Galvus Met) 50/1000 mg once daily, Irbesartan (Aprovel) 300 mg once daily, and Acetylsalicylic Acid (Aspirin) 100 mg once daily.

On admission, the patient’s vital signs were all within the normal ranges, except for decreased blood oxygen saturation, at 85% on room air. The patient had orthopnea and was classified as NYHA III according to clinical findings and reported history. The physical exam revealed grade 2+ lower limb pitting edema and bilateral motor limitation. Of note, the patient had a diabetic ulcer on the dorsum of the left foot and reported a previous spontaneous amputation of the left third toe. The rest of the physical examination concerning the gastrointestinal, urinary, and neurological systems did not reveal any additional features and the patient did not report any other complaints.

**Figure 1:**
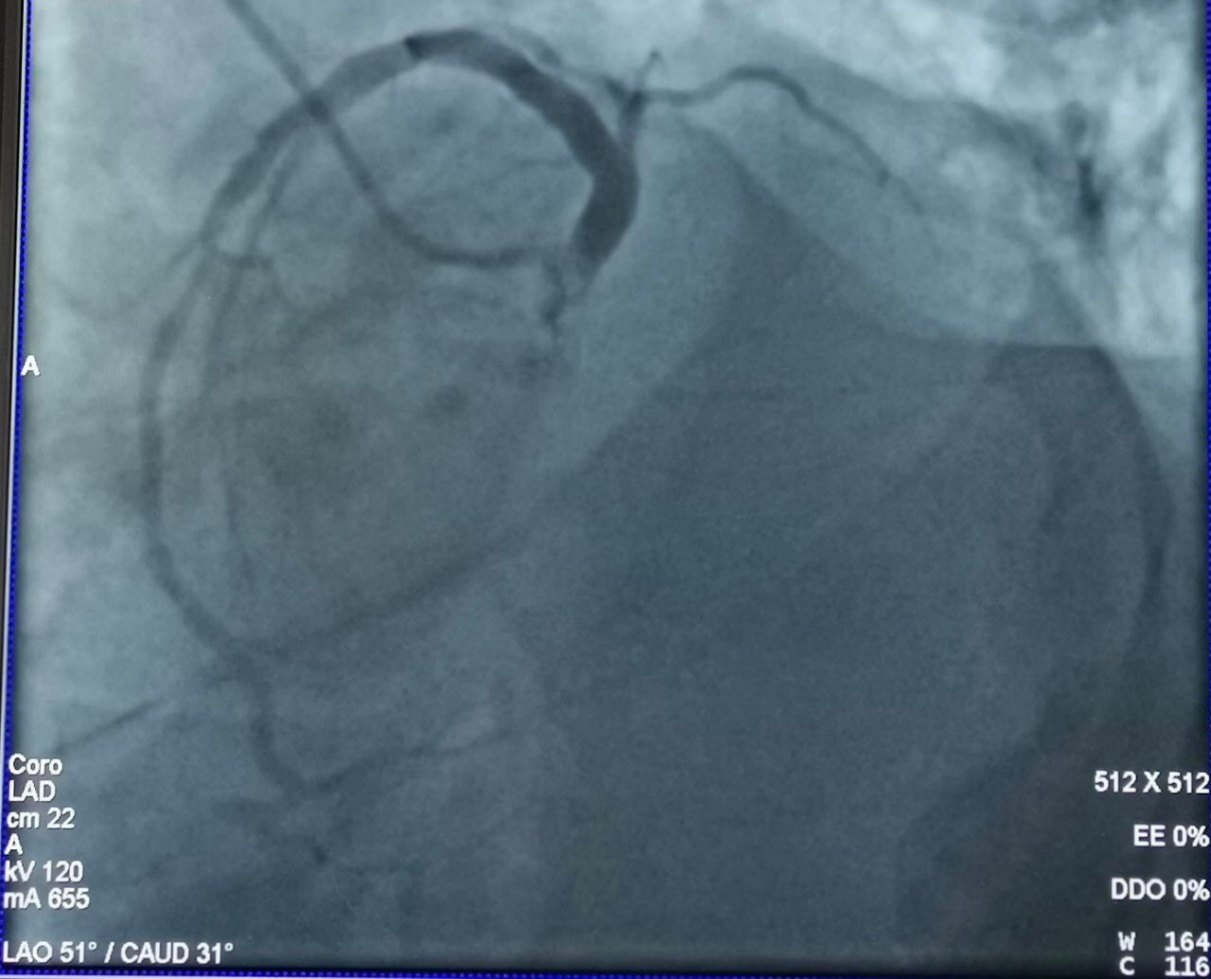
Spider coronary view: Left Anterior Descending (LAD) artery with no origin of circumflex coronary artery.

**Figure 2:**
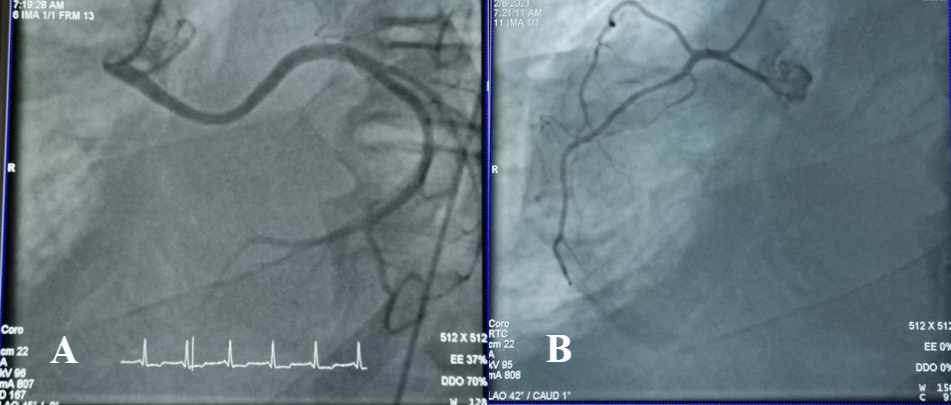
**(A)** Anomalous origin of the circumflex artery from the ostium of the proximal right coronary artery; (**B)** Right Coronary Artery (RCA).

A 12-lead electrocardiogram (ECG) showed T-wave inversion (TWI) in the lateral leads. Computed Tomography (CT) of the chest with intravenous (IV) contrast injection showed bilateral small peripheral emboli in the sub-segmental arteries. It was evident that the patient had moderate to severe bilateral pleural effusion with dilated right heart chambers. Transthoracic echocardiography (TTE) revealed enlarged right heart chambers with pulmonary arterial pressure (PAP) of 90 mmHg. The patient had grade I mitral regurgitation, diastolic dysfunction, and anteroseptal hypokinesia. Ejection fraction (EF) was calculated automatically and was equal to 50%. Arteriography of the lower limbs was normal. Coronary angiography showed that the circumflex artery had an anomalous origin from the proximal ostium of the right coronary artery (Figures 1 and 2).

Laboratory workup showed elevated cardiac enzymes, and the patient was diagnosed with COVID-19-induced myocarditis. The patient was treated with Bisoprolol (Concor) 5 mg once daily, Perindopril Arginine (Coversyl) 5 mg once daily, Empagliflozin (Jardiance) 25 mg once daily, Rivaroxaban (Xarelto) 20 mg once daily, and Furosemide (Lasix) 40 mg IV twice daily. The patient’s condition improved rapidly and he resumed his normal life within one month.

To date, 1½ years later, the patient has been followed-up twice at the clinic, 1 and 6 months after discharge. Repeated laboratory exams were within normal ranges, especially cardiac enzymes, and we didn’t repeat any imaging exams. The patient is taking the same medications at the same doses and living an ordinary life.

## DISCUSSION

In this report, we presented a rare case of a middle-aged man who was admitted to hospital for COVID-19-induced myocarditis in the presence of dyspnea, lower limb edema, and decreased physical activity. An anomalous origin of the left circumflex artery from the right coronary artery was identified later. We will discuss the coronary malformation, of which the attending physician should be aware, to enable correct diagnosis and appropriate treatment.

An anomalous coronary artery is a silent and life-threatening risk factor. Depending on the clinical manifestations, it is possible to distinguish between a malignant variant with an interarterial pathway between the pulmonary artery and aorta, and a benign one with no surrounding arteries.^5^ A cohort study investigating the etiology of the sudden death of 126 military recruits showed that one-third of deaths were caused by the anomalous coronary artery.^6^ However, a case report of three teenagers with anomalous origin of coronary artery showed how malignant the course of the anomaly could be, leading to symptoms like chest pain, syncope, or dizziness with subsequent ventricular fibrillation and heart failure due to prolonged ischemia. All patients required an operation for revascularization and one of them remained symptomatic and was a candidate for heart transplantation.^7^

Brothers et al. suggested that the anomalous arterial anatomy is more prone to dynamic compression and may put the heart at risk of myocardial infarction.^8^ On the other hand, Woudstra et al. indicated that infectious myocarditis causes injury to the cardiac vasculature and subsequent dysregulation of vascular tone including coronary vasospasm.^9^ Consequently, coexistence of these two anomalies, myocarditis and anomalous coronary artery, warranted immediate surgical RCA intervention in an 18-year-old active young man who lived a regular life, but caused the sudden death of a 14-year-old girl.^10^^,^ 11 In contrast, treatment was purely medical in our case, and the patient recovered rapidly within one month without surgical intervention.

In the era of COVID-19, Ghori and Ahmed reported a case of a 34-year-old male referred to the hospital for COVID-19 infection and without cardiac symptoms. However, routine workup revealed a high troponin-T level and left ventricular hypertrophy on ECG.^12^ Echocardiography documented an ejection fraction of 40-45%, and EKG-gated Multidetector Cardiac Computed Tomographic Angiography (MDCCTA) showed an anomalous origin of the left coronary artery from the pulmonary artery. The patient was treated conservatively, as in our case. In contrast, our patient presented one month after COVID-19 infection for myocarditis. Yet, in our case, the patient showed signs of right failure at the same time as the COVID-19 was manifesting. This allows us to question whether COVID19 directly affects the coronary arteries or simply precipitates an already existing disease.

It is worth mentioning that a large prospective cohort study developed an algorithm and used it for decision-making on surgical interventions in anomalous coronary arteries. A total of 44 pediatric patients were treated surgically and showed promising results.^13^ However, technical experience concerning angioplasty of an anomalous left circumflex artery is almost not described in the literature. Angina pectoris, myocardial infarction, and sudden death can be associated with this benign anomaly. Although no ischemic lesions were detectable in this patient, the incidence of this anomaly reflects the possibility of atherosclerotic lesions in this anomalous coronary artery. Reporting this case has made us aware that acute coronary syndrome in the context of myocardial infarction is not the only presentation of an anomalous coronary artery.

In summary, the most common coronary anomaly is an ectopic origin of the left circumflex artery. Management of anomalous circumflex artery is still unclear, especially in cases with coexisting heart disease, such as viral-induced myocarditis. In contrast to the previously described literature, our article is the first to report a case of anomalous circumflex artery with COVID-19-myocarditis in a middle-aged man who only required medical treatment and achieved satisfactory results. According to this report, we can postulate that diagnosis of an anomalous coronary artery and choice of treatment modality are not easy tasks and require experienced and skilled physicians.
